# Make me more comfortable: effects of a hypnosis session on pain perception in chronic pain patients

**DOI:** 10.3389/fpsyg.2024.1362208

**Published:** 2024-02-28

**Authors:** David Ogez, Mathieu Landry, Rémi Caron-Trahan, Anne-Eva Jusseaux, Maryse Aubin, Jade Véronneau, Valentyn Fournier, Nadia Godin, Moulay Idrissi, Pierre Rainville, Philippe Richebé

**Affiliations:** ^1^Research Centre, Hôpital Maisonneuve-Rosemont, Montréal, QC, Canada; ^2^Department of Anesthesiology and Pain Medicine, Université de Montréal, Montréal, QC, Canada; ^3^Département de Psychologie, Université de Montréal, Montréal, QC, Canada; ^4^SCALab – Sciences Cognitives et Sciences Affectives, Université de Lille, Lille, France; ^5^Faculty of Dental Medicine, Université de Montréal, Montréal, QC, Canada; ^6^Research Centre, Institut Universitaire de Gériatrie de Montréal, Montréal, QC, Canada

**Keywords:** self-hypnosis, chronic pain, anxiety, quality of life, intervention program, pre-post study

## Abstract

**Introduction:**

Approximately one-quarter of Canadians experience chronic pain, a debilitating condition often necessitating opioid use, which raises concerns regarding dependency and overdose risks. As an alternative, we developed the HYlaDO program (Hypnose de la Douleur, hypnosis of Pain in French), a novel self-hypnosis approach for chronic pain management. The development of this program followed the ORBIT model, a comprehensive framework for designing interventions encompassing several phases ranging from design to efficacy assessment.

**Methods:**

In the present work, we conducted a preliminary evaluation of the HYlaDO program with 21 participants (18 of the 21 patients were included in the analysis). The primary objective was to determine one session of the program’s effectiveness in altering pain, anxiety and relaxation via pre-post analysis. The secondary goal was to examine the long-term effects across the same measures, in addition to the overall quality of life.

**Results:**

The results highlight the benefits of our approach, while participants reported short-term significant pain reduction, decreased anxiety, and increased relaxation. Additionally, preliminary trends suggest improvements in physical activity and quality of life metrics.

**Discussion:**

These positive outcomes highlight HYlaDO’s potential as an alternative to opioid therapy for chronic pain. Encouraged by these results, we aim to extend our research to a broader and more diverse cohort, paving the way for comprehensive randomized controlled trials. This expansion will further validate HYlaDO’s efficacy and its role in transforming chronic pain management.

## Introduction

In Canada, chronic pain is a prevalent issue, affecting more than 7 million individuals, or about one in four, over their lifetimes (Canadian pain task force, 2019). This condition not only impacts personal health but also imposes a considerable economic strain. The total cost, including both direct expenses such as medical services and indirect costs like lost productivity, is approximately $40 billion (Canadian pain task force, 2019). At the level of individual, chronic pain significantly affects mental health, quality of life, and social inclusion among Canadians. Most notably, between 35 and 60% of those suffering from chronic pain is at an elevated risk of developing anxiety disorders (Choinière et al., 2020). Social consequences are also evident, with chronic pain contributing to early disability and impairing both personal and professional lives (Mills et al., 2019). In Canada, these social and health deficits are exacerbated by the lack of readily available services and delays in accessing specialized care (Canadian pain task force, 2019). The average wait time for pain management clinics is between 8 and 10 months, allowing ample time for pain to become chronic. These prolonged waits increase levels of pain and distress, while also reducing the likelihood of successful therapy. In parallel the lack of services, effective treatments, and lengthy wait times yield increased usage of potent analgesics, such as opioids. This heightened usage results in tolerance and dependence, limiting their availability in pain clinics in a timely manner (Mills et al., 2019). In sum, the current situation regarding chronic pain in Canada is difficult and is likely to get worse due aging population.

Following this adverse context, the Canadian government mandated a group of specialists, the Pain Task Force, to guide decision-makers in enhancing chronic pain prevention and management strategies (Canadian pain task force, 2020). A key recommendation from this panel was to increase the adoption of non-pharmacologic interventions, such as hypnosis, mindfulness, and acceptance therapy. This recommendation followed from strong empirical supporting their effectiveness in pain management. Furthermore, focused on cultivating pain self-management skills, these interventions present innovative solutions to address issues like healthcare accessibility, substance misuse, and the escalation of severe pain-related outcomes. Research consistently demonstrates that these strategies are effective in mitigating the risk of chronic pain persistence and its associated comorbidities. They integrate well within multimodal and biopsychosocial treatment frameworks, significantly benefiting patients’ mental health and overall quality of life (Langlois et al., 2022).

Among these approaches, research indicates that clinical hypnosis represents an efficient non-pharmacological intervention for pain management in various clinical populations suffering from chronic pain (Langlois et al., 2022). Hypnotic interventions stand out for their ability to maintain its effects over an extended period of time based on a procedure that can simply reinstate suggestions for analgesia (Houzé et al., 2021). Moreover, in addition to pain reduction, evidence shows that clinical hypnosis can also reduce anxiety, improve sleep, and enhance quality of life of patients (Thompson et al., 2019). In summary, hypnosis represents a viable non-pharmacological treatment option for chronic pain (Jensen et al., 2006, 2020).

In contrast to hetero-hypnosis, which involves the guidance of a clinician, self-hypnosis is characterized by the patient’s performance of hypnotic induction and suggestion procedures (Hammond, 2001). This approach promotes self-management of chronic pain outside therapeutic sessions with a healthcare professional (Langlois et al., 2022). Self-hypnosis relies on two key elements: instructions for practicing self-hypnosis and audio recordings (Bicego et al., 2021; Eaton et al., 2021). Despite its apparent effectiveness, self-hypnosis training remains largely unexplored, with limited information in the literature about the optimal method for providing self-hypnosis instructions (Milling et al., 2021; Samami et al., 2021). The present work aims to address this lacuna by further developing a new standardized program specifically tailored for self-hypnosis training in chronic pain management.

## Objectives

**Figure 1:**
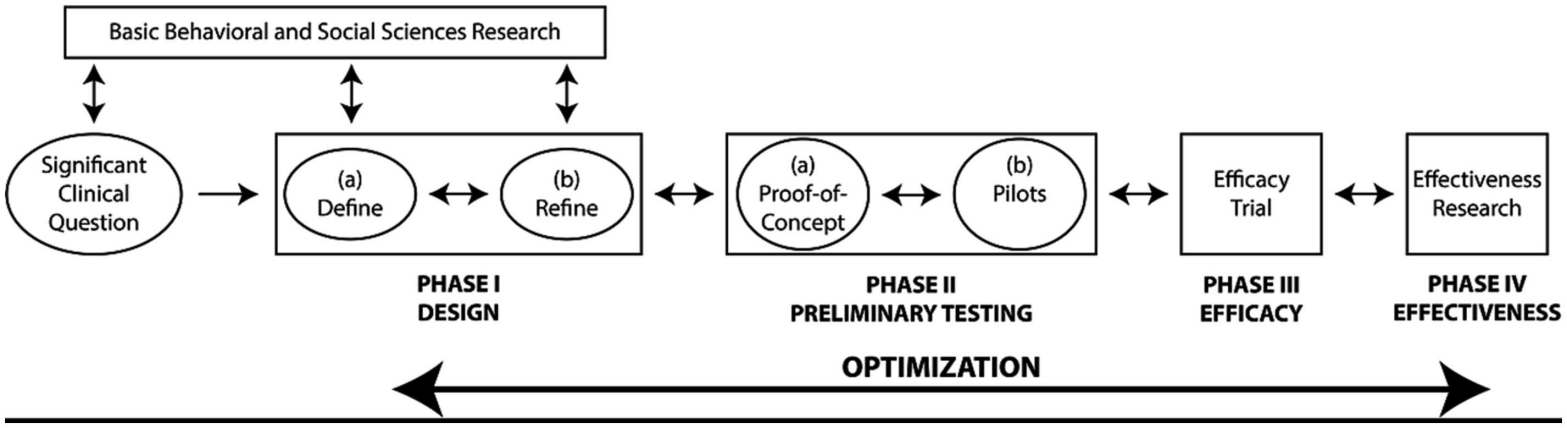
ORBIT model, figure adapted from Czajkowski et al. (2015).

This research introduces a self-hypnosis training program, developed based on insights from prior chronic pain studies. Our approach to designing this program followed the ORBIT framework (Figure 1; Czajkowski et al., 2015). The ORBIT model represents a flexible overarching framework to guide and optimize the development of behavioral treatment across four distinct phases. During phase I, the program is conceptualized and refined, incorporating feedback and suggestions from potential users to ensure its relevance and efficacy. Phase II involves conducting preliminary studies to assess the program’s initial impact and to set the stage for more extensive research. The subsequent phases, III and IV, are dedicated to rigorous efficacy and effectiveness studies, respectively. These phases are crucial for establishing the program’s validity and determining its practical applicability in real-world settings. Based on this framework, the current study present work that was done during phase II. In this regard, the aim of the present work is to evaluate the effects of this program’s hypnosis techniques in the context of chronic pain. Our evaluation is twofold: first, we aim to assess the immediate impact of a single hypnosis session on participants’ levels of pain intensity, anxiety, and relaxation; second, our goal is also to examine the long-term benefits of ongoing self-hypnosis practice on the same measure and overall quality of life. We hypothesize that regular self-hypnosis will significantly improve the quality of life for these individuals, alongside marked reductions in anxiety and pain. This hypothesis is predicated on the notion that self-hypnosis can effectively modulate pain perception and bolster coping strategies, thus positively influencing both mental and physical health outcomes.

## Materials and methods

### HYlaDO intervention program

HYlaDO (“HYpnose pour la DOuleur”; hypnosis for pain in French) is a self-hypnosis program designed for improving the quality of life of chronic pain patients. As we mentioned previously, the design of our program tracks the stages of the ORBIT model. Our previous research documents the outcome of Phases Ia and Ib (Caron-Trahan et al., 2023a,b). The initial version of the HYlaDO program comprised eight sessions: a session introducing the participants, a session introducing hypnosis, 5 sessions offering 5 heterohypnosis exercises and recommendations for self-hypnosis practice, and a session for conclusion and feedback. The five exercises aimed at emotional release, acceptance, pain modification, pain reducing with magic glove, and confidence building. Each exercise followed a structured pattern, including a hypnotic induction procedure, a deepening phase, specific hypnotic work depending on the session objective, post-hypnotic suggestions aiming to maintain effects and facilitate self-hypnosis practice, and a guided return to wakefulness. The training process was supported by seven weekly video conferences led by a professional hypnotherapist who guided participants through these exercises. Additionally, participants had access to recordings of the exercises and practical self-hypnosis guidelines. They were also invited to participate in weekly videoconference practice groups to reproduce each of these hypnosis exercises.

### Participants and procedures

We conducted a pre-post non-randomized study using HYlaDO version 1.0. This study was carried out simultaneously with the refinement study and is part of phase II of the ORBIT model (Figure 1) – i.e., preliminary studies. The current research included 21 out of 32 patients that were trained in self-hypnosis for reducing pain between June 2020 and April 2021. The inclusion criteria were established as follows: at least 18 years old, experiencing chronic pain, receiving treatment at the hospital’s pain clinic, have previously participated in the self-hypnosis training program within the last year, and consent to participate in this research study. There were no exclusion criteria for this study since the patients were selected during the clinical phase and therefore met the criteria for training in hypnosis techniques. To participate in this clinical intervention, patients had to understand French and not have any disorders that would impair communication (too much cognitive impairment, severe psychosis).

**Figure 2:**
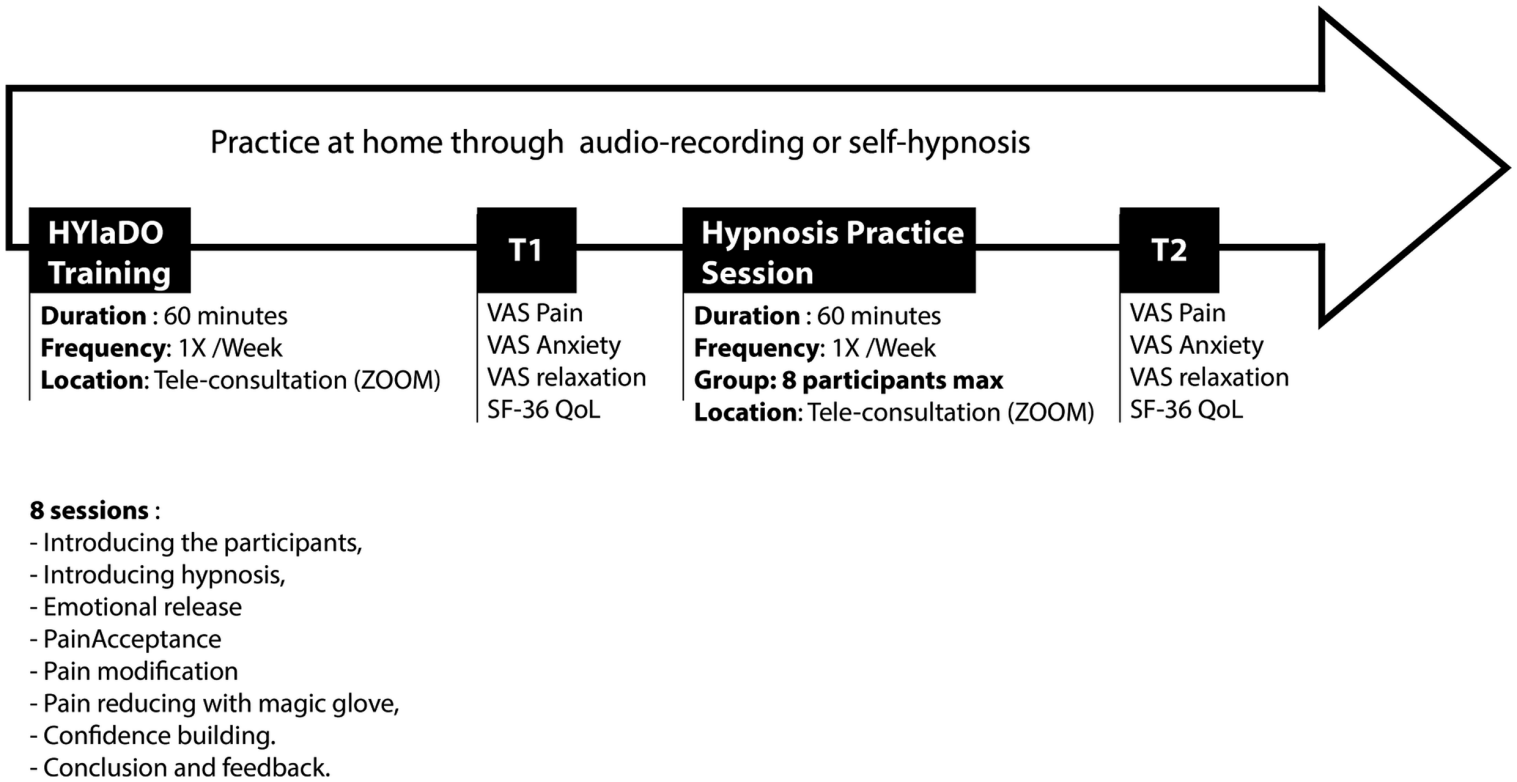
Study protocol.

Participants were recruited by invitation from a research assistant that was independent from the clinical provider. Interested individuals were presented with a consent form to sign during their hospital visit. Following consent, 18 of the 21 participants engaged in the study at two key time points: The commencement of the research (T1) and 6 months later (T2). At T1, participants completed a brief socio-demographic and clinical questionnaire, along with scales measuring pain, anxiety, relaxation, and quality of life. This was followed by a 30-min hypnosis session for relaxation and acceptance of pain (exercise from HYlaDO session 3), after which participants re-evaluated their levels of pain, anxiety, and relaxation. They were then instructed to practice self-hypnosis regularly, using options such as audio-recordings from the program, independent practice, and the weekly videoconferencing sessions offered to them. Twenty-four sessions of self-hypnosis practice were carried out during the 6 months between these measurement times. The second assessment at T2 involved a similar procedure. Participants returned to the clinic to reassess their pain, anxiety, relaxation, and quality of life through the same scales. This was accompanied by another 30-min hypnosis session, after which they again rated their pain, anxiety, and relaxation levels. The study protocol is summarized in Figure 2.

The mean age of patients was 52 (SD = 11) years old. Fifteen of them were women, and 6 were men. In term of occupation, 2 were unemployed, 3 in temporary work interruption, 3 were retired, 5 in invalidity, 1 was a part-time employee, and 7 were full-time employees. The detailed sociodemographic and medical characteristics are presented in Table 1.

**Table 1:** Descriptive data.

	*N* = 21	%
Sex		
Female	15	71
Male	6	29
Age (years)		
Mean (SD)	52 (11)	
Marital status		
Single	6	29
Divorced	4	19
Relationship	11	52
Having children		
Yes	17	81
No	4	19
Education		
Secondary	7	33
College	2	10
University	12	57
Professional status		
Unemployed	2	10
Temporary	3	14
Retired	3	14
Invalidity	5	24
Part-time work	1	5
Full-time work	7	33

### Measures

The primary outcome of this study was pain intensity, assessed using a visual analog scale (VAS) with 11 points ranging from 0 (representing “no pain”) to 10 (indicating “worst imaginable pain”) (Thong et al., 2018). Secondary outcomes included anxiety, relaxation, and quality of life. Anxiety levels were measured using a similar VAS, with 0 signifying “no anxiety” and 10 representing “extreme anxiety.” Relaxation was assessed pre- and post-practice using a VAS ranging from 0 (“not at all relaxed”) to 10 (“highly relaxed”). These three parameters were evaluated before and after each hypnosis session at both T1 and T2. Quality of life was assessed using the 36-Item Short Form Survey (SF-36) at T1 and T2, prior to the hypnosis practices. The SF-36 encompasses 36 items across eight domains: physical activity limitations due to health issues, social activity limitations due to physical or emotional problems, usual role limitations due to physical health, bodily pain, general mental health, role limitations due to emotional problems, vitality, and overall health perception (Lins and Carvalho, 2016). Scores from these domains are aggregated using a specific scoring key, yielding a composite quality of life score ranging from low to high [α(T1) = 0.952; α(T2) = 0.887]. Additionally, two component scores are calculated: a Physical Component Summary [α(T1) = 0.916; α(T2) = 0.840] and a Mental Component Summary [α(T1) = 0.914; α(T2) = 0.936], providing a nuanced overview of participants’ quality of life.

### Analyses

Socio-demographic data were analyzed descriptively. Perceived pain, anxiety, and relaxation scores were compared at different times: pre- and post- hypnosis sessions at both T1 and T2, and between T1 and T2 for pre- and post-hypnosis time points, respectively. The difference in pain, anxiety, and relaxation scores between T1 and T2 were also compared. Also, total quality of life scores and sub-scores were compared between T1 and T2. Two-tailed Wilcoxon signed-rank test was used given the repeated measures design and the small sample size. Statistical significatively threshold were settled at α = 0.05. All analyses were run using SPSS 28.0.1. software.

## Results

**Figure 3:**
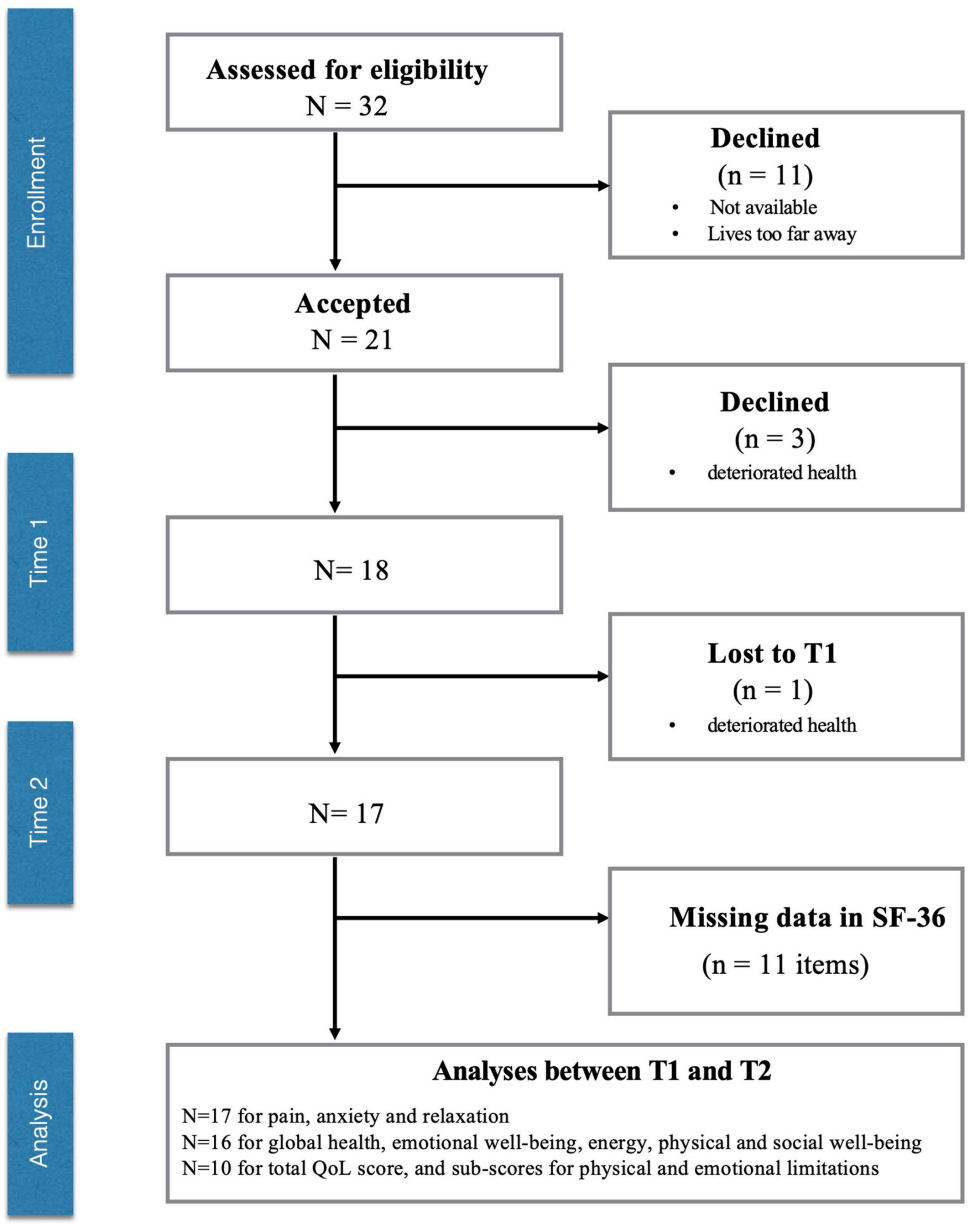
Flowchart.

As illustrated in the flowchart (Figure 3), 18 of the 32 participants successfully completed the measurements in our study. Dropouts were due to participants’ unavailability, logistical challenges such as residing too far from the laboratory, and the worsening of physical health conditions.

### Perceived pain, anxiety, and relaxation

For perceived pain and anxiety scores, non-parametric paired comparisons were conducted with Wilcoxon tests to compare the scores before and after the hypnosis sessions at T1 and T2. Results show a significant difference between the scores on the variables of interest before and after hypnosis session at T1 with lower scores of perceived pain [*W*(18) = −3.366; *p* < 0.001] and anxiety [*W*(18) = −2.955; *p* = 0.003], and higher score of relaxation [*W*(18) = 2.996; *p* = 0.003]. The same pattern is observed for measures before and after hypnosis at T2 with lower score of perceived pain [*W*(17) = −3.415; *p* < 0.001] and anxiety [*W*(17) = −3.18; *p* = 0.001], and higher scores of relaxation [*W*(17) = 3.638; *p* < 0.001].

**Figure 4:**
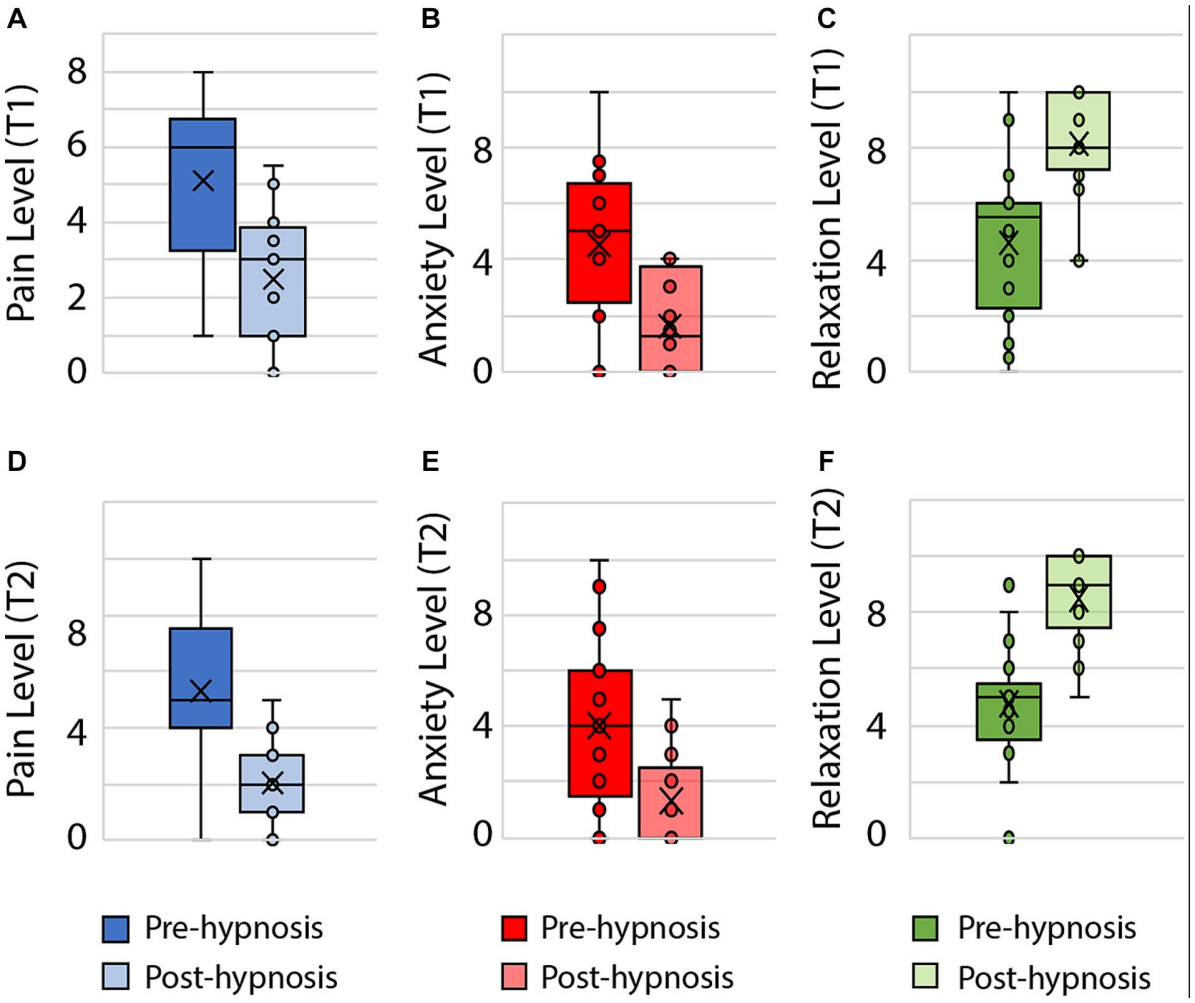
Significant changes in pain, anxiety and relaxation levels for pre- and post-hypnosis across T1 and T2. **(A)** Pain level (T1), **(B)** Anxiety level (T1), **(C)** Relaxation level (T1), **(D)** Pain level (T2), **(E)** Anxiety level (T2), **(F)** Relaxation level (T2).

No significant difference is observed between the scores of perceived pain before hypnosis session at T1 and T2 [*W*(17) = 0.095; *p* = 0.925], neither is for anxiety scores [*W*(17) = −1.177; *p* = 0.239]. The comparison of scores after hypnosis sessions between T1 and T2 does not show significant results for perceived pain [*W*(17) = −1.166; *p* = 0.243] or anxiety [*W*(17) = −0.820; *p* = 0.412] (Figure 4).

### Quality of life

Total scores for quality of life and sub-scores on the SF-36 were compared between T1 and T2. Analyses reveal no significant difference between the total scores of quality of life at T1 and T2 [*W*(10) = 1.125; *p* = 0.260], neither for different sub-scores such as physical limitation [*W*(10) = 1.350; *p* = 0.177], emotional limitation [*W*(10) = 1.382; *p* = 0.167], emotional well-being [*W*(16) = 0.739; *p* = 0.460], pain [*W*(16) = 0.09; *p* = 0.929], and global health [*W*(16) = −0.751; *p* = 0.452]. When considered as a whole, mental quality of life (i.e., sum of limitations in social activities and usual activities because emotional problem, general mental health and vitality sub-scores) did not differ significantly between T1 and T2 [*W*(10) = 0.969; *p* = 0.333]. However, statistical tendency is observed for several sub-scores such as physical functioning [*W*(16) = 1.728; *p* = 0.084], social functioning [*W*(16) = 1.667; *p* = 0.095], and energy [*W*(16) = 1.657; *p* = 0.097]. When computed as such (i.e., sum of limitations in social activities and usual activities because physical problem, body pain and general perception of health sub-scores), the dimension of physical quality of life tends to be different between T1 and T2 [*W*(10) = 1.682; *p* = 0.093].

## Discussion

This study aimed to evaluate the short- and long-term benefits of HYlaDO, a self-hypnosis approach, on reducing pain and anxiety levels, increasing relaxation level, and improving quality of life in patients with chronic pain. This work was done in the context of the preliminary test phase (Phase II) of the ORBIT model. The results confirmed that the HYlaDO program improves the perception of pain, anxiety and relaxation. Furthermore, we also observed improvements at 6-month follow-up on quality-of-life sub-scores for some individuals (*N* = 11). Our findings regarding the effects of a hypnosis session from the HYlaDO program in pre-post-intervention represents a critical step in the development of a non-pharmacological approach in pain clinical practice. Already, previous research highlights the efficacy of hypnosis in pain management, as demonstrated in numerous fundamental and clinical studies (Lang et al., 2000; Mills et al., 2019; Thompson et al., 2019). Consistent with this body work, our program led to a reduction in perceived pain and anxiety, two central targets among patients suffering from chronic pain, would indicate that these patients can experience a sense of physical and emotional comfort without pharmacological interventions. It is further complemented by the patients’ ability to achieve relaxation during the hypnosis session, demonstrating their capacity to regain control over their bodies, often perceived as beyond their control.

Our research corroborates previous findings in chronic pain management, which underline the crucial role of self-care skills in patient treatment (Jensen et al., 2020; Langlois et al., 2022). Our study builds on this work by harnessing patients’ endogenous abilities to enhance their well-being, thereby promoting their autonomy. However, our analysis did not show significant differences in the assessments conducted between T1 and T2, with a six-month interval between these measurements. Several reasons may account for the absence of this effect. First, we noted pronounced improvements in self-reported pain perception, anxiety, and relaxation at the session-level. The substantial benefits observed within a single session suggest that improvements across sessions may only be marginal, indicative of a ceiling effect for these measures. Still, the consistency in results between T1 and T2 suggests that participants were at least able to maintain these improvements over time, underscoring the sustained impact of the intervention.

Secondly, as a preliminary study, our limited sample size constrained our capacity to detect anything but large effect sizes. Despite this, we noted improvements in quality of life for some individuals (*N* = 11) between T1 and T2. Quality of life encompasses various factors, divided into emotional and physical sub-scales (Lins and Carvalho, 2016). This possible improvement is specific to the physical sub-scale of the SF-36, which relates to physical functioning, social functioning, and energy. A more extensive sample size in future research would enable a more accurate estimation of effect sizes and provide the statistical power necessary to evaluate these potential benefits more thoroughly. Conversely, the emotional sub-scale of the SF-36, which includes distress and the general perception of one’s health. It seems unlikely that a brief intervention, like the one we are proposing here, can swiftly address the complexity of these patients’ mental fragility in such a short timeframe. For example, chronic pain conditions lead to significant socio-professional and financial consequences, such as a loss of time at work and reduced financial contributions. Therefore, the absence of an effect across T1 and T2 for this subscale is hardly surprising.

Third, the participants in this study were treated at a pain clinic, and we did not document the proposed treatments in a detailed manner. We could not isolate these treatments either due to our small sample size. For future studies, we will document and introduce them as variables in our analyses.

Lastly, inter-individual variability in hypnotic responding represent another aspects, a fundamental yet frequently neglected aspect of hypnotic phenomena in clinical settings (Houzé et al., 2021). Such variability can stem from diverse factors including psychological background, individual susceptibility to hypnosis, and previous experiences with hypnotic techniques (REFs). This variability implies that individuals react distinctly to identical suggestions (Houzé et al., 2021). Unfortunately, in the context of our study, we did not collect specific information regarding this variability in hypnotic susceptibility among participants. Consequently, our analysis lacks an exploration of how these individual differences in response to hypnosis might have played a role in the outcomes observed. This limitation is significant, as understanding the extent to which hypnotic susceptibility influences therapeutic outcomes could provide valuable insights for tailoring hypnotic interventions more effectively. Future research in this domain should aim to incorporate measures of hypnotic susceptibility to better assess its impact on clinical results. This approach could potentially lead to more personalized and effective therapeutic strategies in the application of hypnotherapy.

In sum, this preliminary study confirmed that a single session from the HYlaDO program benefits chronic pain patients along several dimensions. However, we could not confirm the benefits of the program between T1 and T2. Considering the milestones outlined in the ORBIT model, this justifies advancing to the next stage, a pilot randomized controlled study, aimed at testing our protocol and gathering data. This step will enable us to estimate effect sizes and calculate the sample size required for a future clinical trial (ORBIT III). Ultimately, if this project proves effective, it could be widely offered in pain clinics as a non-pharmacological approach based on hypnosis.

## Data availability statement

The raw data supporting the conclusions of this article will be made available by the authors, without undue reservation.

## Ethics statement

The studies involving humans were approved by Maisonneuve-Rosemont Hospital Ethics (CER No: 2022-2741). The studies were conducted in accordance with the local legislation and institutional requirements. The participants provided their written informed consent to participate in this study.

## Author contributions

DO: Conceptualization, Formal analysis, Funding acquisition, Methodology, Project administration, Supervision, Validation, Writing – original draft, Writing – review & editing. ML: Formal analysis, Validation, Writing – review & editing. RC-T: Formal analysis, Investigation, Writing – review & editing. A-EJ: Investigation, Writing – review & editing. MA: Conceptualization, Funding acquisition, Writing – review & editing. JV: Writing - review & editing. VF: Formal analysis, Validation, Writing – review & editing. NG: Writing – review & editing. MI: Formal analysis, Validation, Writing – review & editing. PRa: Writing – review & editing. PRi: Resources, Writing – review & editing.
